# Supplementary material for: Fire air pollution reduces global terrestrial productivity
Source: Nat Commun. 2018 Dec 21;9:5413. doi: 10.1038/s41467-018-07921-4 (PMC6303378; doi:10.1038/s41467-018-07921-4)
Supplement: Supplementary file 1 — Supplementary Information [file 41467_2018_7921_MOESM1_ESM.pdf]

Supplementary Information

Fire air pollution reduces global terrestrial productivity

Xu Yue and Nadine Unger

Supplementary Table 1 | Summary of simulations for fire pollution effects

Simulations	Model	Input	O_3 effect [a]	Output
GC_NOFIRE	GEOS-Chem	All emissions without fires	N/A	Monthly aerosol and hourly [O_3]
GC_FIRE	GEOS-Chem	All emissions with fires	N/A	Monthly aerosol and hourly [O_3]
CRM_NA	CRM	No aerosols	N/A	Hourly direct and diffuse PAR
CRM_AA	CRM	Aerosols from GC_NOFIRE	N/A	Hourly direct and diffuse PAR
CRM_FA	CRM	Aerosols from GC_FIRE	N/A	Hourly direct and diffuse PAR
YIBS_NA	YIBs	PAR from CRM_NA	None	Monthly GPP
YIBS_AA	YIBs	PAR from CRM_AA	None	Monthly GPP
YIBS_FA	YIBs	PAR from CRM_FA	None	Monthly GPP
YIBS_NOH	YIBs	PAR from CRM_NA<br>[O_3] from GC_NOFIRE	High	Monthly GPP
YIBS_NOL	YIBs	PAR from CRM_NA<br>[O_3] from GC_NOFIRE	Low	Monthly GPP
YIBS_FOH	YIBs	PAR from CRM_NA<br>[O_3] from GC_FIRE	High	Monthly GPP
YIBS_FOL	YIBs	PAR from CRM_NA<br>[O_3] from GC_FIRE	Low	Monthly GPP
YIBS_AAOH	YIBs	PAR from CRM_AA<br>[O_3] from GC_NOFIRE	High	Monthly GPP
YIBS_AAOL	YIBs	PAR from CRM_AA<br>[O_3] from GC_NOFIRE	Low	Monthly GPP
YIBS_FAOH	YIBs	PAR from CRM_FA<br>[O_3] from GC_FIRE	High	Monthly GPP
YIBS_FAOL	YIBs	PAR from CRM_FA<br>[O_3] from GC_FIRE	Low	Monthly GPP

[a] Ozone damage applied in the simulation can be low or high for the same level of [O_3], depending on the selection of damaging coefficients [1].

Supplementary Table 2 | Sensitivity experiments for O_3 damaging scheme with YIBs model

ID	Simulations [a]	$[O_3]$ (ppbv)	O_3 effect	GPP (Pg)	ΔGPP (%) [b]
1	YIBS_O000	0	Null	138.4	0
2	YIBS_O020L	20	Low	137.3	-0.8
3	YIBS_O040L	40	Low	132.2	-4.5
4	YIBS_O060L	60	Low	125.6	-9.3
5	YIBS_O080L	80	Low	119.4	-13.8
6	YIBS_O100L	100	Low	113.8	-17.8
7	YIBS_O120L	120	Low	108.9	-21.3
8	YIBS_O140L	140	Low	104.5	-24.5
9	YIBS_O160L	160	Low	100.6	-27.4
10	YIBS_O020H	20	High	135.1	-2.4
11	YIBS_O040H	40	High	123.4	-10.9
12	YIBS_O060H	60	High	111.3	-19.6
13	YIBS_O080H	80	High	101.4	-26.8
14	YIBS_O100H	100	High	93.3	-32.6
15	YIBS_O120H	120	High	86.6	-37.4
16	YIBS_O140H	140	High	81.0	-41.5
17	YIBS_O160H	160	High	76.2	-44.9

[a] Each simulation is performed for 2007-2011 using the same meteorology. The last 3 years are used to calculate the O_3-induced damages to GPP as shown in Fig. 1.
[b] Compared with GPP in simulation YIBS_O000.

Supplementary Table 3 | Sensitivity experiments for fire ozone effects with YIBs model

**Simulations** [a]	**Ozone input**	**O_3 effect** [b]	**GPP (Pg C yr^{-1})**
YIBS_NOH_LE	GC_NOFIRE	High	130.3
YIBS_NOL_LE	GC_NOFIRE	Low	133.5
YIBS_FOH_LE	GC_FIRE	High	129.2
YIBS_FOL_LE	GC_FIRE	Low	133.0

[a] Each simulation is performed for 2001-2011.
[b] Ozone damaging coefficients of evergreen broadleaf forest (EBF) are lower by 1/3 for both high and low sensitivities.

Supplementary Table 4 | Sensitivity experiments for cloud diffuse effects on GPP using CRM model

ID	Simulations [a]	Cloud scale [b]	Total PAR ($W\ m^{-2}$)	Diffuse fraction
1	CRM_C000	0	128.1	0.10
2	CRM_C001	0.1	127.9	0.10
3	CRM_C002	0.2	127.1	0.12
4	CRM _C003	0.3	125.6	0.15
5	CRM_C004	0.4	123.5	0.19
6	CRM_C005	0.5	120.9	0.23
7	CRM_C006	0.6	117.9	0.27
8	CRM_C007	0.7	114.7	0.31
9	CRM_C008	0.8	111.3	0.35
10	CRM_C009	0.9	107.8	0.38
11	CRM_C010	1.0	104.3	0.41
12	CRM_C012	1.2	98.4	0.45
13	CRM_C014	1.4	93.0	0.48
14	CRM_C017	1.7	86.1	0.51
15	CRM_C020	2.0	80.3	0.52
16	CRM_C030	3.0	66.6	0.54
17	CRM_C040	4.0	58.1	0.54
18	CRM_C050	5.0	52.3	0.53
19	CRM_C070	7.0	44.9	0.50
20	CRM_C100	10.0	38.6	0.46

[a] Each simulation is performed for 2007-2011 without aerosol radiative effects. The CRM model output of hourly diffuse and direct PAR radiation is used as input for YIBs model.
[b] Observed 3-D cloud fraction and liquid water path are multiplied by corresponding scaling factors.

Supplementary Table 5 | Sensitivity experiments for cloud diffuse effects on GPP using YIBs model

ID	Simulations [a]	PAR input	GPP (Pg)	ΔGPP (%) [b]
1	YIBS_C000	CRM_C000	106.3	0
2	YIBS_C001	CRM_C001	107.1	0.8
3	YIBS_C002	CRM_C002	110.5	3.9
4	YIBS_ C003	CRM _C003	115.9	9.1
5	YIBS_C004	CRM_C004	122.3	15.0
6	YIBS_C005	CRM_C005	128.3	20.7
7	YIBS_C006	CRM_C006	133.3	25.4
8	YIBS_C007	CRM_C007	136.8	28.7
9	YIBS_C008	CRM_C008	138.7	30.5
10	YIBS_C009	CRM_C009	139.2	31.0
11	YIBS_C010	CRM_C010	138.4	30.2
12	YIBS_C012	CRM_C012	135.0	27.0
13	YIBS_C014	CRM_C014	129.8	22.1
14	YIBS_C017	CRM_C017	120.9	13.8
15	YIBS_C020	CRM_C020	112.0	5.4
16	YIBS_C030	CRM_C030	87.1	-18.1
17	YIBS_C040	CRM_C040	69.8	-34.3
18	YIBS_C050	CRM_C050	57.9	-45.6
19	YIBS_C070	CRM_C070	43.3	-59.3
20	YIBS_C100	CRM_C100	32.1	-69.8

[a] Each simulation is performed for 2007-2011 using hourly diffuse and direct PAR from the corresponding CRM simulation in Supplementary Table 4.
[b] Compared with GPP in simulation YIBS_C000.

Supplementary Table 6 | Sensitivity experiments for fire aerosol effects with CRM model

Simulations [a]	Aerosols	Cloud [b]	Total PAR ($W\ m^{-2}$)	Diffuse fraction
CRM_AA_C00	GC_NOFIRE	Cloud fraction and LWP set to zero	123.6	0.164
CRM_AA_C05	GC_NOFIRE	Cloud fraction and LWP reduce by 50%	117.0	0.282
CRM_AA_C20	GC_NOFIRE	Cloud fraction and LWP increase by 100%	77.9	0.559
CRM_AA_C3H	GC_NOFIRE	3-hourly cloud variables are applied	92.0	0.468
CRM_FA_C00	GC_FIRE	Cloud fraction and LWP set to zero	123.2	0.169
CRM_FA_C05	GC_FIRE	Cloud fraction and LWP reduce by 50%	116.6	0.286
CRM_FA_C20	GC_FIRE	Cloud fraction and LWP increase by 100%	77.6	0.562
CRM_FA_C3H	GC_FIRE	3-hourly cloud variables are applied	91.7	0.471
CRM_FA_BC05	Fire BC reduces by 50%	Observed cloud fraction and LWP	100.9	0.455
CRM_FA_BC20	Fire BC increases by 100%	Observed cloud fraction and LWP	100.7	0.455
CRM_FA2	All fire aerosols increase by 100%	Observed cloud fraction and LWP	100.5	0.458
CRM_FA3	All fire aerosols increase by 200%	Observed cloud fraction and LWP	100.2	0.461

[a] Each simulation is performed for 2001-2011. The model output of hourly diffuse and direct PAR radiation is used as input for YIBs model.
[b] Cloud fraction and liquid water path (LWP) change at the same rate.

Supplementary Table 7 | Sensitivity experiments for fire aerosol effects with YIBs model

Simulations [a]	PAR input	GPP (Pg C yr^{-1})
YIBS_AA_C00	CRM_AA_C00	112.2
YIBS_AA_C05	CRM_AA_C05	129.8
YIBS_AA_C20	CRM_AA_C20	109.9
YIBS_AA_C3H	CRM_AA_C3H	125.4
YIBS_FA_C00	CRM_FA_C00	112.9
YIBS_FA_C05	CRM_FA_C05	130.2
YIBS_FA_C20	CRM_FA_C20	109.7
YIBS_FA_C3H	CRM_FA_C3H	125.4
YIBS_FA_BC05	CRM_FA_BC05	136.8
YIBS_FA_BC20	CRM_FA_BC20	136.6
YIBS_FA2	CRM_FA2	136.7
YIBS_FA3	CRM_FA3	136.7

[a] Each simulation is performed for 2001-2011 using hourly diffuse and direct PAR from the corresponding CRM simulation in Supplementary Table 6. Ozone damaging effects are turned off.

Supplementary Table 8 | Sensitivity experiments for evaluation of modeling uncertainties

Runs	Descriptions	Changes in GPP
O3_L	O3 damaging with low sensitivity	YIBS_FOL – YIBS_NOL
O3_H	O3 damaging with high sensitivity	YIBS_FOH – YIBS_NOH
LE_L	O3 damaging with low sensitivity coefficient of EBF reduces by 1/3	YIBS_FOL_LE – YIBS_NOL_LE
LE_H	O3 damaging with high sensitivity coefficient of EBF reduces by 1/3	YIBS_FOH_LE – YIBS_NOH_LE
CLD05	Observed cloud fraction is scaled by 0.5	YIBS_FA_C05 – YIBS_AA_C05
CLD10	Observed daily cloud fraction	YIBS_FA – YIBS_AA
CLD3H	Observed 3-hour cloud fraction	YIBS_FA_C3H – YIBS_AA_C3H
CLD20	Observed cloud fraction is scaled by 2	YIBS_FA_C20 – YIBS_AA_C20
BC05	BC content reduces by 50% OC content increases by 5%	YIBS_FA_BC05 – YIBS_AA
BC20	BC content increases by 100% OC content reduces by 10%	YIBS_FA_BC20 – YIBS_AA
FA2	Fire aerosol concentrations are doubled	YIBS_FA2 – YIBS_AA
FA3	Fire aerosol concentrations are tripled	YIBS_FA3 – YIBS_AA

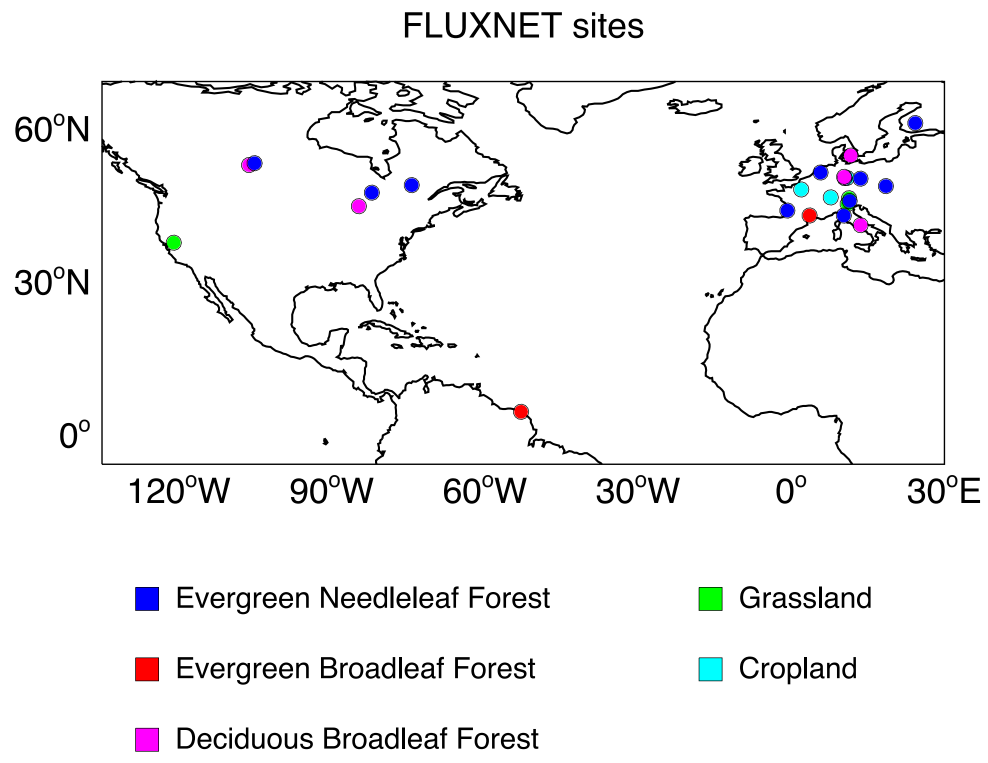

Supplementary Figure 1 | Distribution of 24 FLUXNET sites with diffuse radiation. Sites are selected with at least 5-year measurements. The color indicates different land types as evergreen needleleaf forest (ENF, blue), evergreen broadleaf forest (EBF, red), deciduous broadleaf forest (DBF, magenta), grasslands (GRA, green), and croplands (CRO, cyan). These sites provide half-hourly observations of diffuse radiation.

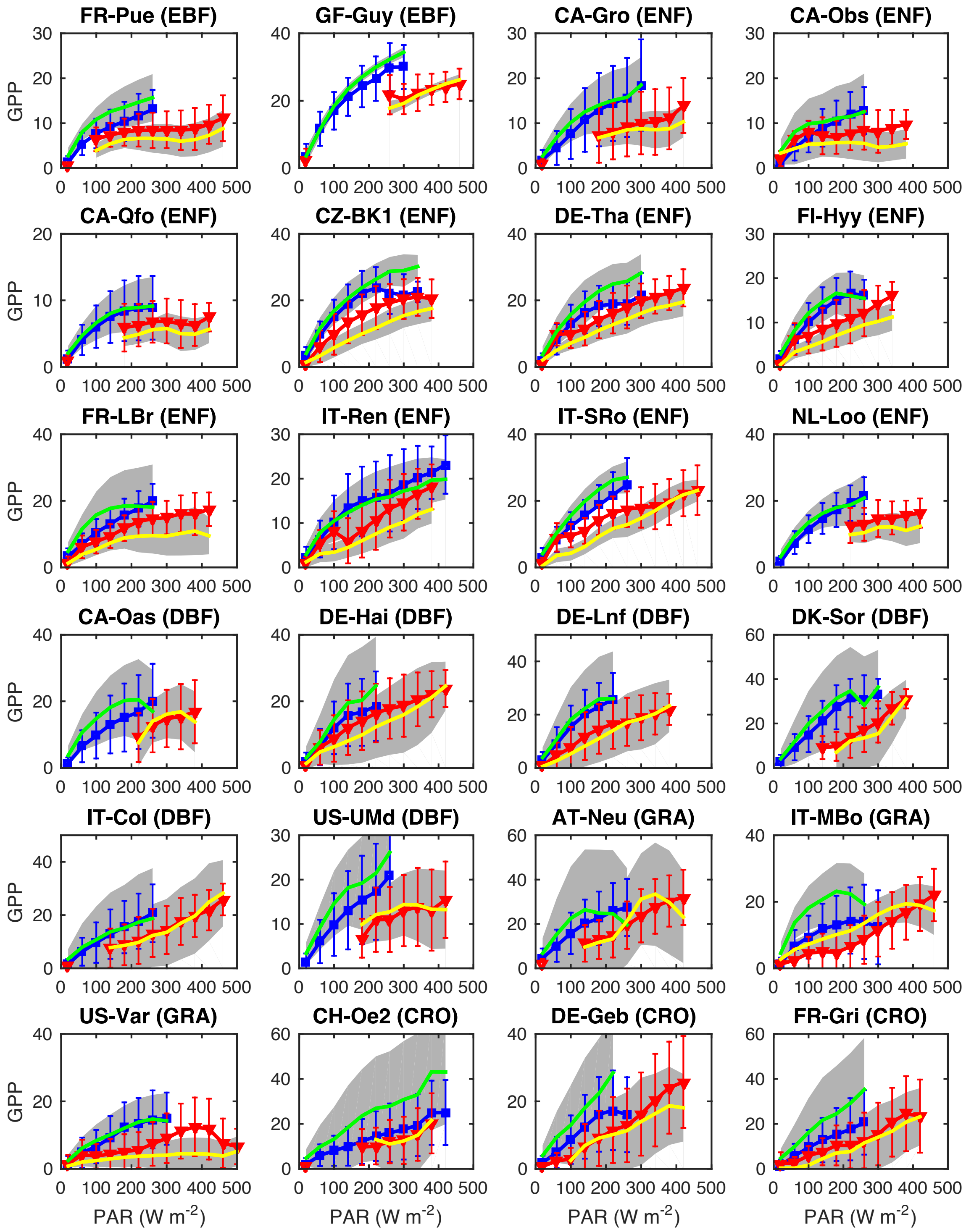

Supplementary Figure 2 | Evaluation of simulated GPP responses to direct and diffuse radiation. The comparisons are performed at 24 FLUXNET sites. For each site, observations are split into 'diffuse' and 'direct' conditions if the diffuse fraction is >0.8 (blue squares) and <0.2 (red triangles), respectively. The categorized observations are then averaged over PAR bins of 40 W m^{-2} with error bars indicating one standard deviation of GPP for each bin. Similarly, simulations are split into 'diffuse' (green) and 'direct' (yellow) bins of PAR and GPP is calculated for each bin with gray shading indicating one standard deviation. The name of site and the specific vegetation type is listed on the title of each panel.

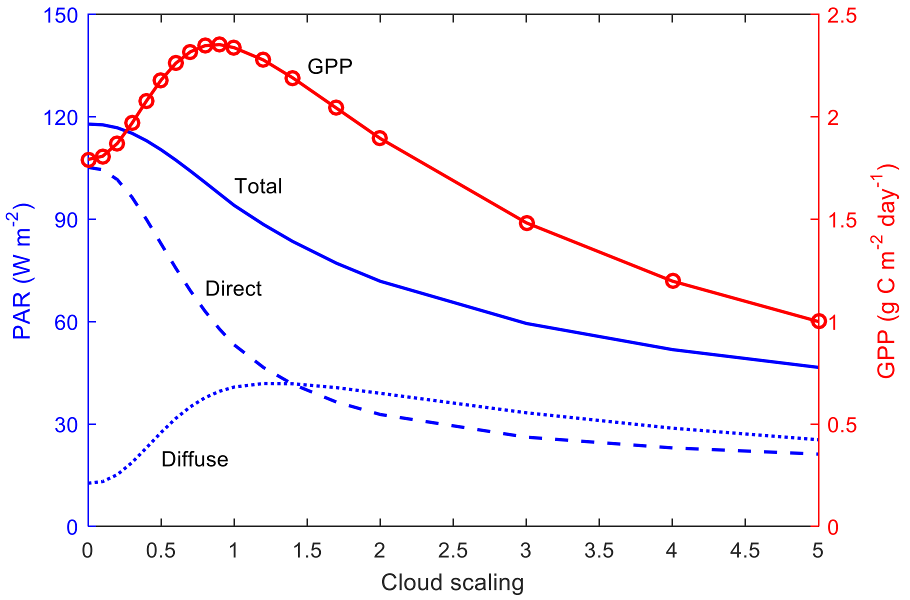

Supplementary Figure 3 | Responses of global GPP to varied cloud scalings. Results shown are global GPP (red) and PAR radiation (blue) for different simulations with varied cloud scaling factor (Supplementary Table 4).

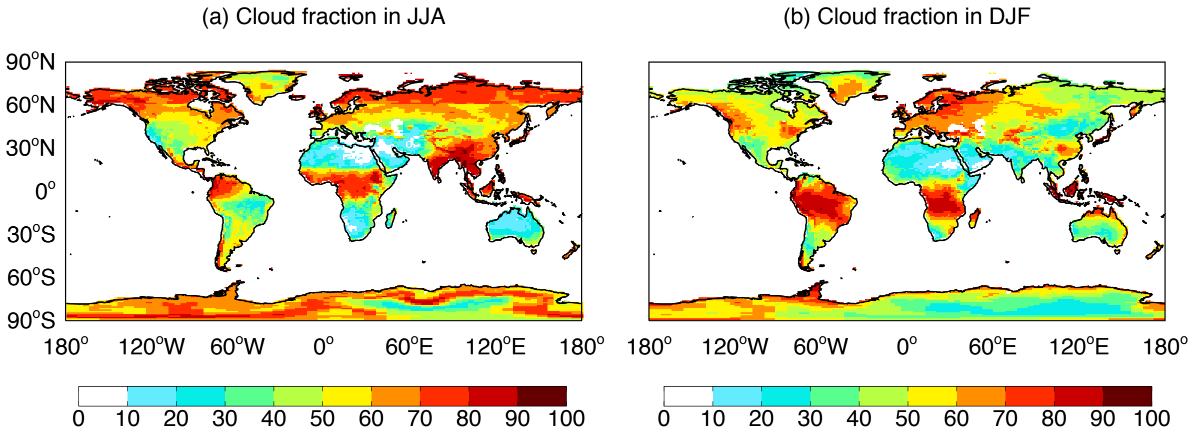

Supplementary Figure 4 | Seasonality of global cloud amount. Results shown are cloud fraction (%) in (a) June-July-August and (b) December-January-February from the SYN1deg product of NASA Clouds and the Earth's Radiant Energy System (CERES)[2].

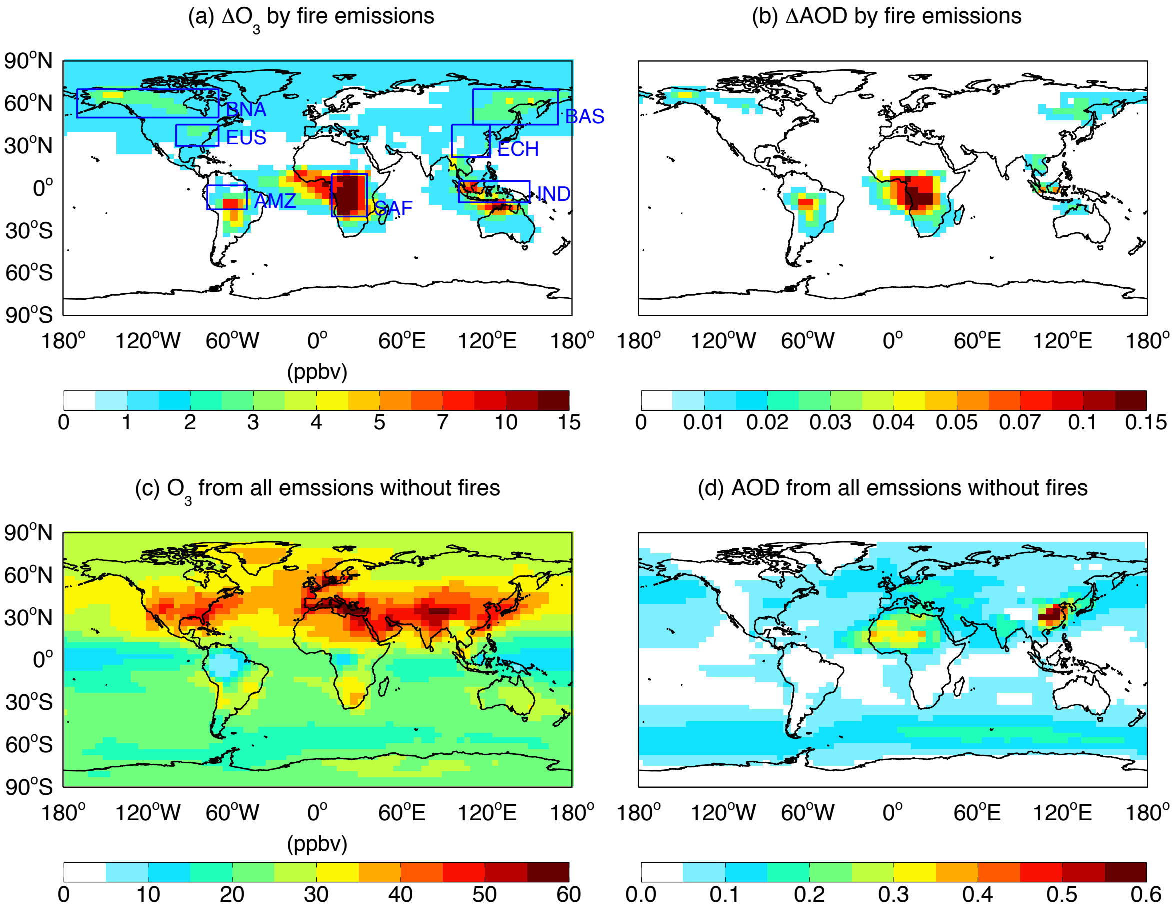

Supplementary Figure 5 | Ozone and aerosol optical depth (AOD) from fire and non-fire sources. Results shown are the annual mean (a, c) surface O_3 concentration and (b, d) AOD at 550 nm caused by (a, b) fire emissions alone (GC_FIRE – GC_NOFIRE) and (c, d) non-fire sources (GC_NOFIRE). For (a) and (b), only significant ($p < 0.1$) changes are shown. The color scales are different between the top and bottom panels. Seven regions in Figure (a) are Amazon (AMZ), southern Africa (SAF), Indonesia (IND), eastern U.S. (EUS), eastern China (ECH), boreal North America (BNA), and boreal Asia (BAS).

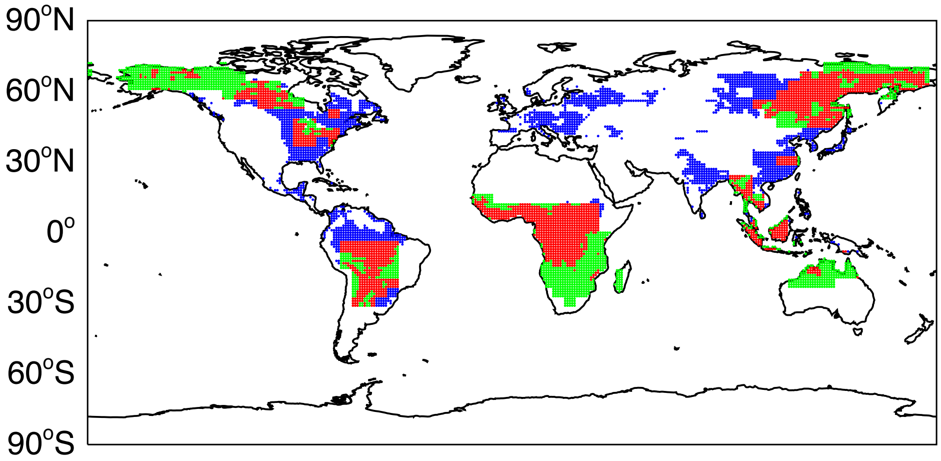

Supplementary Figure 6 | Changes in GPP caused by fire O_3 from different sources. For each land grid, fire O_3-induced changes in GPP (ΔGPP) and fire O_3 concentrations ([O_3]) are derived and sorted, respectively. Grids with both high ΔGPP (>80th percentile) and high fire [O_3] (>80th percentile) are marked with red. Grids with high ΔGPP (>80th percentile) without high fire [O_3] (<80th percentile) are marked with blue. Grids with high fire [O_3] (>80th percentile) but relatively small ΔGPP (<80th percentile) are marked with green. For red patches, fire [O_3] is high and the resultant ΔGPP is large. For blue patches, low to medium fire [O_3] results in large ΔGPP. For green patches, high fire [O_3] results in small ΔGPP.

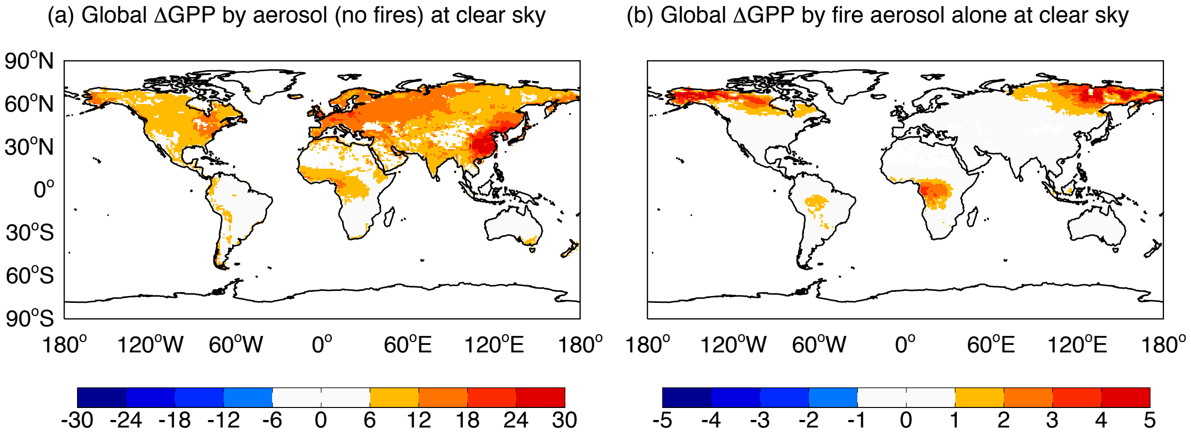

Supplementary Figure 7 | Percentage change in GPP caused by aerosol diffuse fertilization effect at clear sky. The effects of aerosol from all sources (anthropogenic + natural) except fire emissions are shown in the left, and those from fire emissions alone are in the right. Units: %.

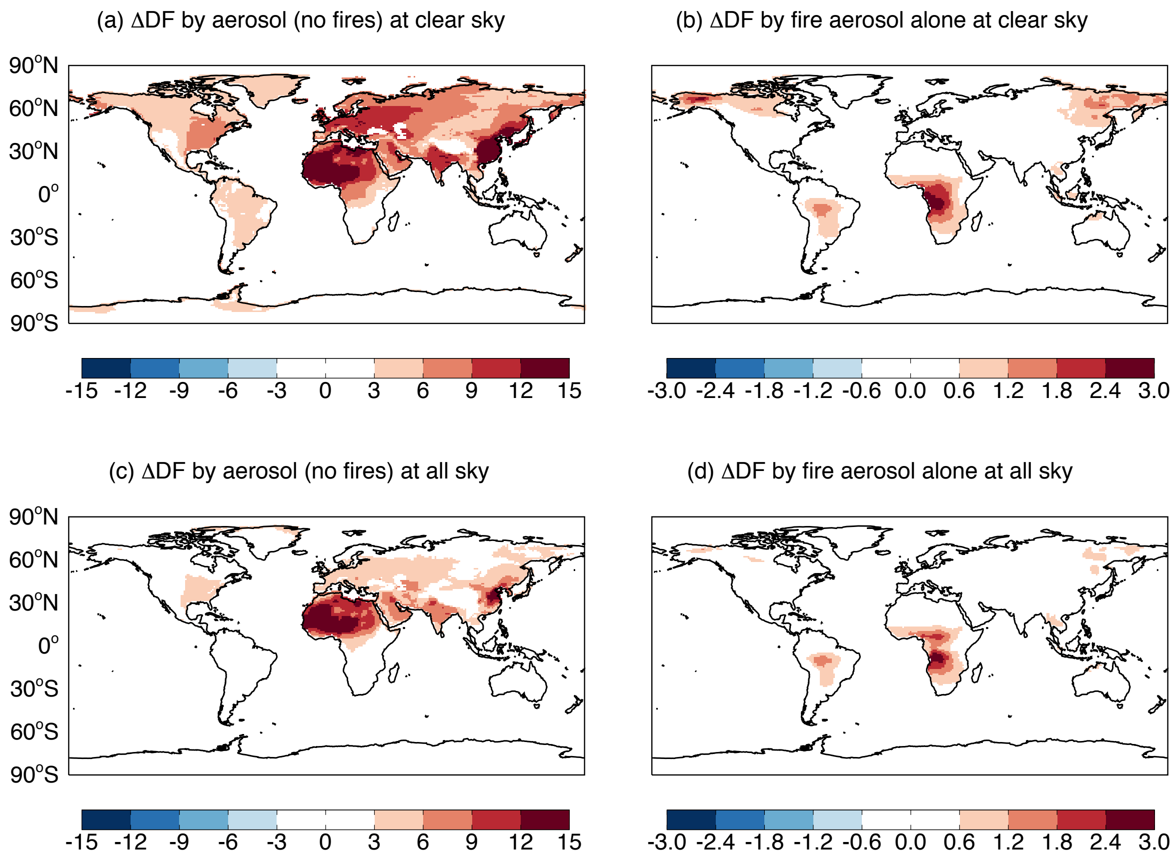

Supplementary Figure 8 | Changes in diffuse fraction caused by aerosols. Results shown are the changes in diffuse fraction (diffuse/total) due to (a, c) all aerosols without fire emissions and (b, d) fire aerosols alone at the (a, b) clear-sky and (c, d) all-sky conditions. Units: %.

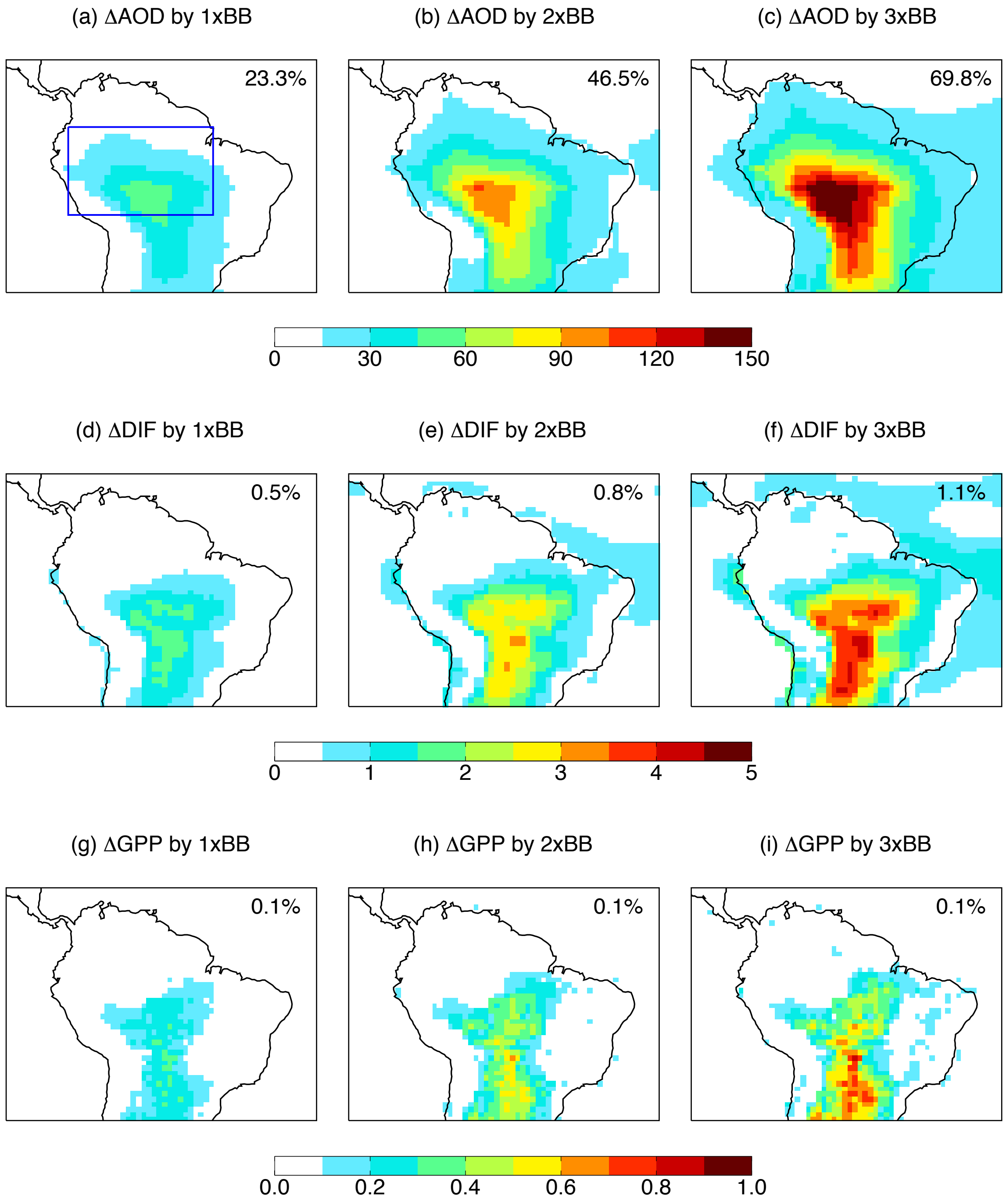

Supplementary Figure 9 | Predicted changes in diffuse fraction and GPP due to enhanced fire aerosols. Results shown are percentage changes in (a, b, c) AOD, (d, e, f) diffuse radiation, and (g, h, i) GPP over Amazon region due to (a, d, g) current, (b, e, h) double, and (c, f, i) triple fire aerosols.

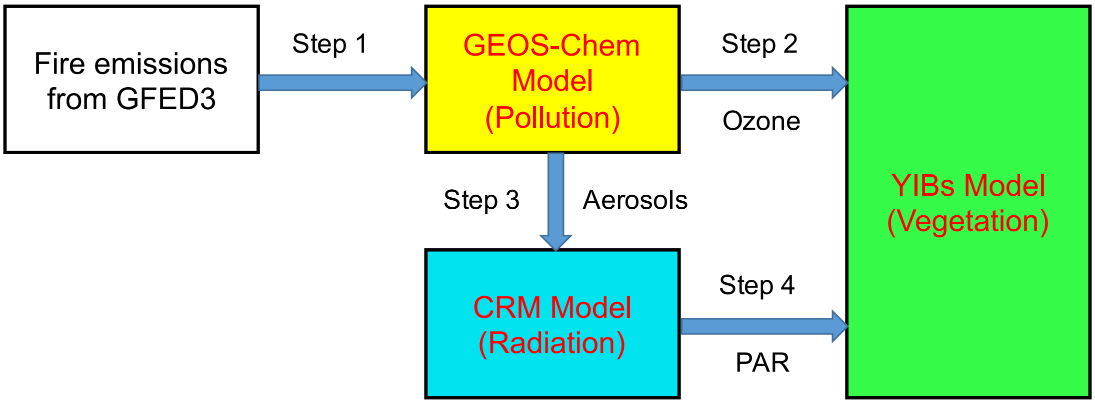

Supplementary Figure 10 | Models and simulations used for this study. Step 1: Simulate fire-induced aerosols and ozone using GEOS-Chem Model for 2001-2011. Step 2: Simulate ozone damaging to GPP using YIBs model. Step 3: Simulate aerosol-induced PAR radiation change using CRM model. Step 4: Simulate GPP responses to PAR change using YIBs model.

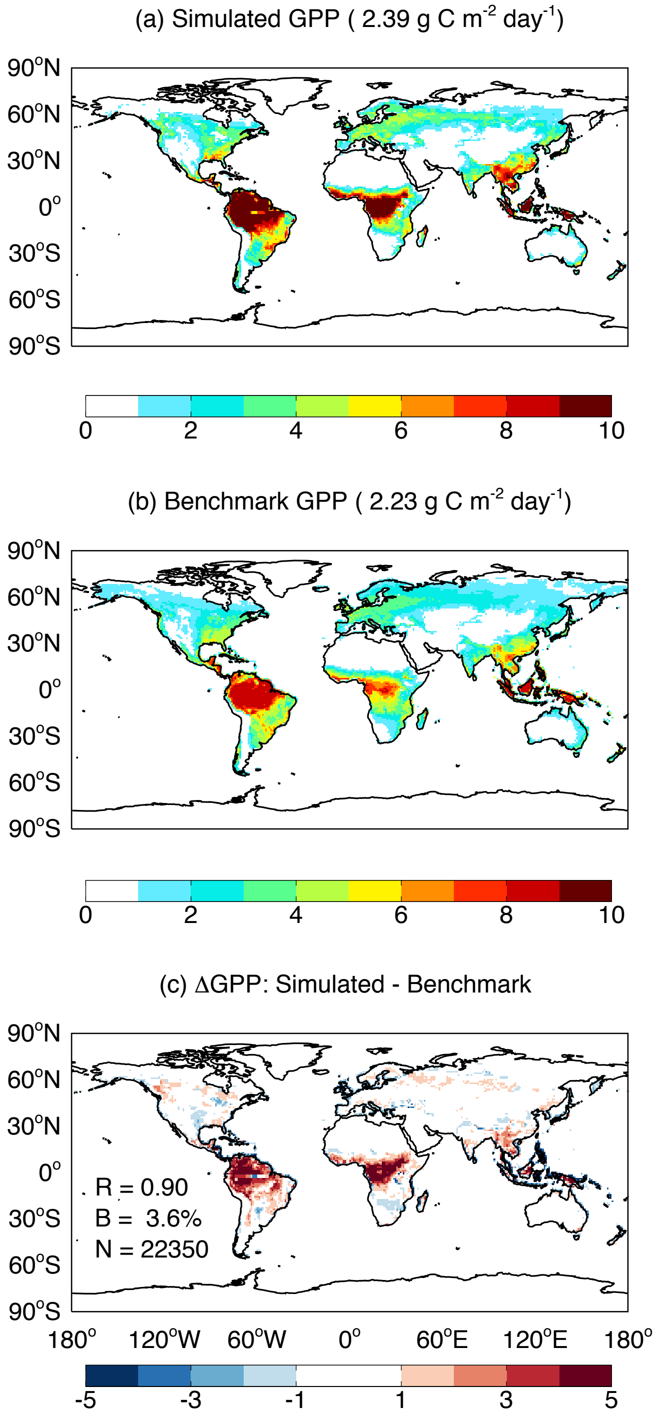

Supplementary Figure 11 | Evaluation of simulated gross primary productivity (GPP) by YIBs model. Simulation (a) is performed using the YIBs vegetation model, which is driven with hourly 1°× 1° meteorological forcings for 2001-2011 from the WATCH Forcing Data methodology applied to ERA-Interim reanalysis data (WFDEI)[3]. Global areal weighted GPP are shown on the title brackets. Benchmark product (b) is upscaled from FLUXNET using regression trees[4]. The differences between simulation and benchmark product is shown in (c). The correlation coefficient (R) and relative biases (B) are shown in the bottom figures with indication of total land grid number (N) used for statistics.

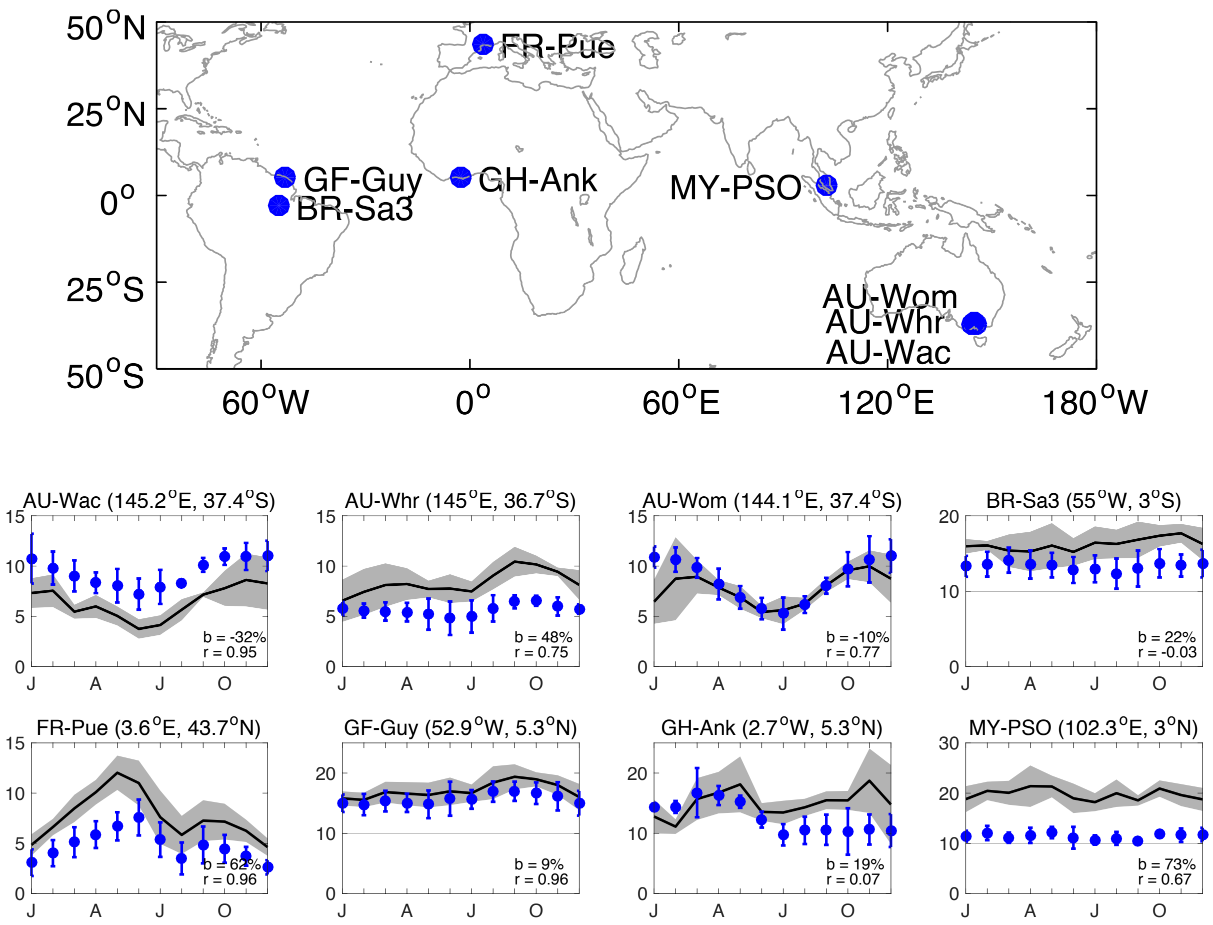

Supplementary Figure 12 | Evaluation of simulated GPP at evergreen broadleaf forest (EBF) sites. In the top panel, 8 EBF sites with at least four-year measurements from FLUXNET network (http://fluxnet.fluxdata.org) are selected. In the bottom panels, both simulations and observations are averaged for each individual site over the period when measurements are available. Error bars represent one standard deviation of observations while shadings represent that of simulations. The relative bias and correlation coefficient are shown on each panel.

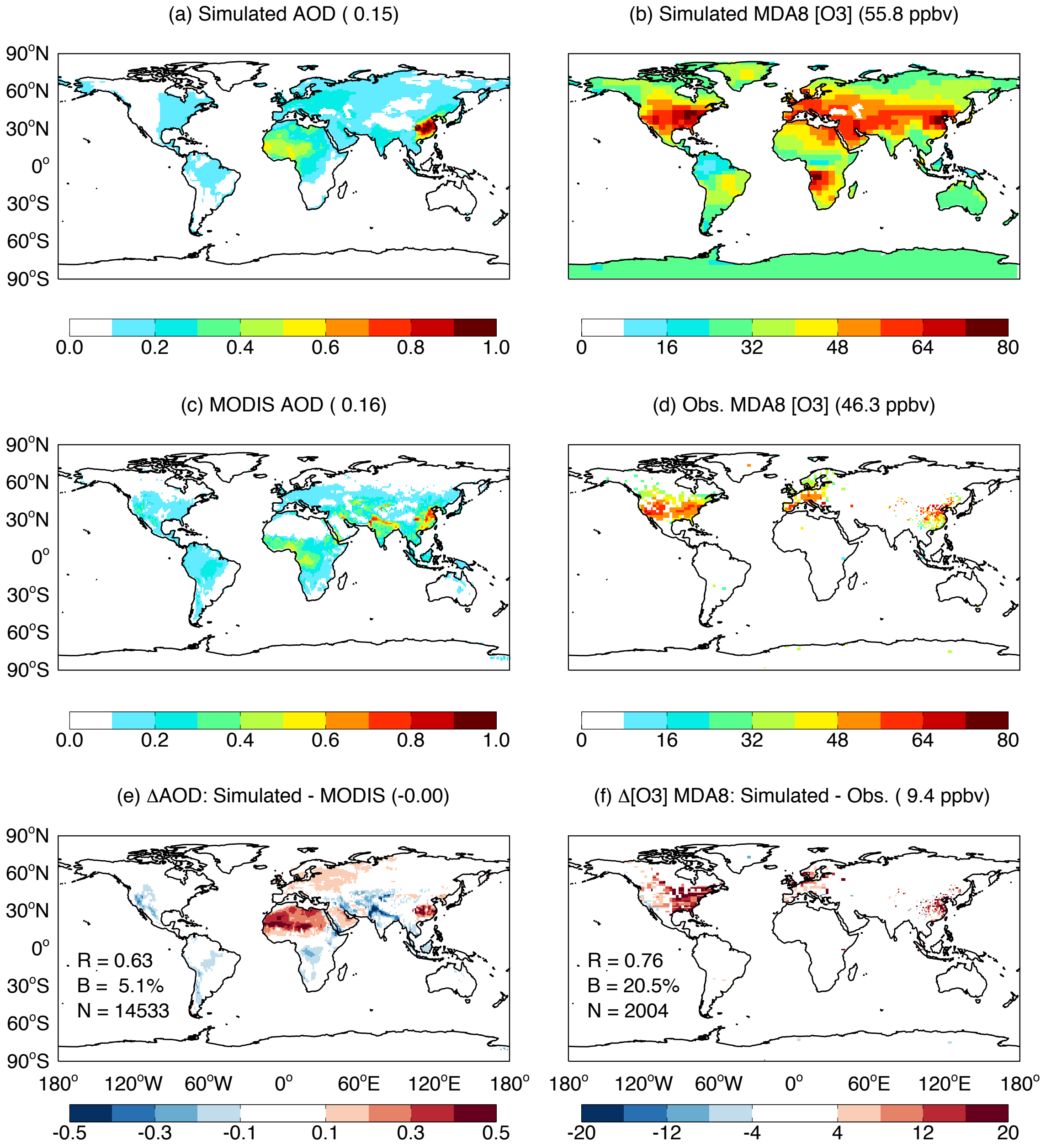

Supplementary Figure 13 | Evaluation of simulated air pollution by GEOS-Chem model. Results shown are the (a, c) annual aerosol optical depth (AOD) at 550 nm and (b, d) summertime maximum daily 8-h average (MDA8) O_3 concentrations from (a, b) simulations, (c, d) observations, and (e, f) their differences averaged for period of 2001-2011. Simulations are performed with the GEOS-Chem chemical transport model, which is driven with MERRA meteorology and emissions from anthropogenic, biogenic, and biomass burning sources. Observed AOD is retrieved from the Moderate Resolution Imaging Spectroradiometer (MODIS, https://modis.gsfc.nasa.gov). Surface MDA8 O_3 over North America and Europe is adopted from the Global Gridded Surface Ozone Dataset compiled by Sofen, et al. [5]. O_3 over China is interpolated from data at ~1500 sites operated by the Ministry of Environmental Protection (http://english.mep.gov.cn). Global areal weighted AOD and MDA8 O_3 are shown on the title brackets. The correlation coefficient (R) and relative biases (B) are shown in the bottom figures with indication of grid numbers (N) used for statistics.

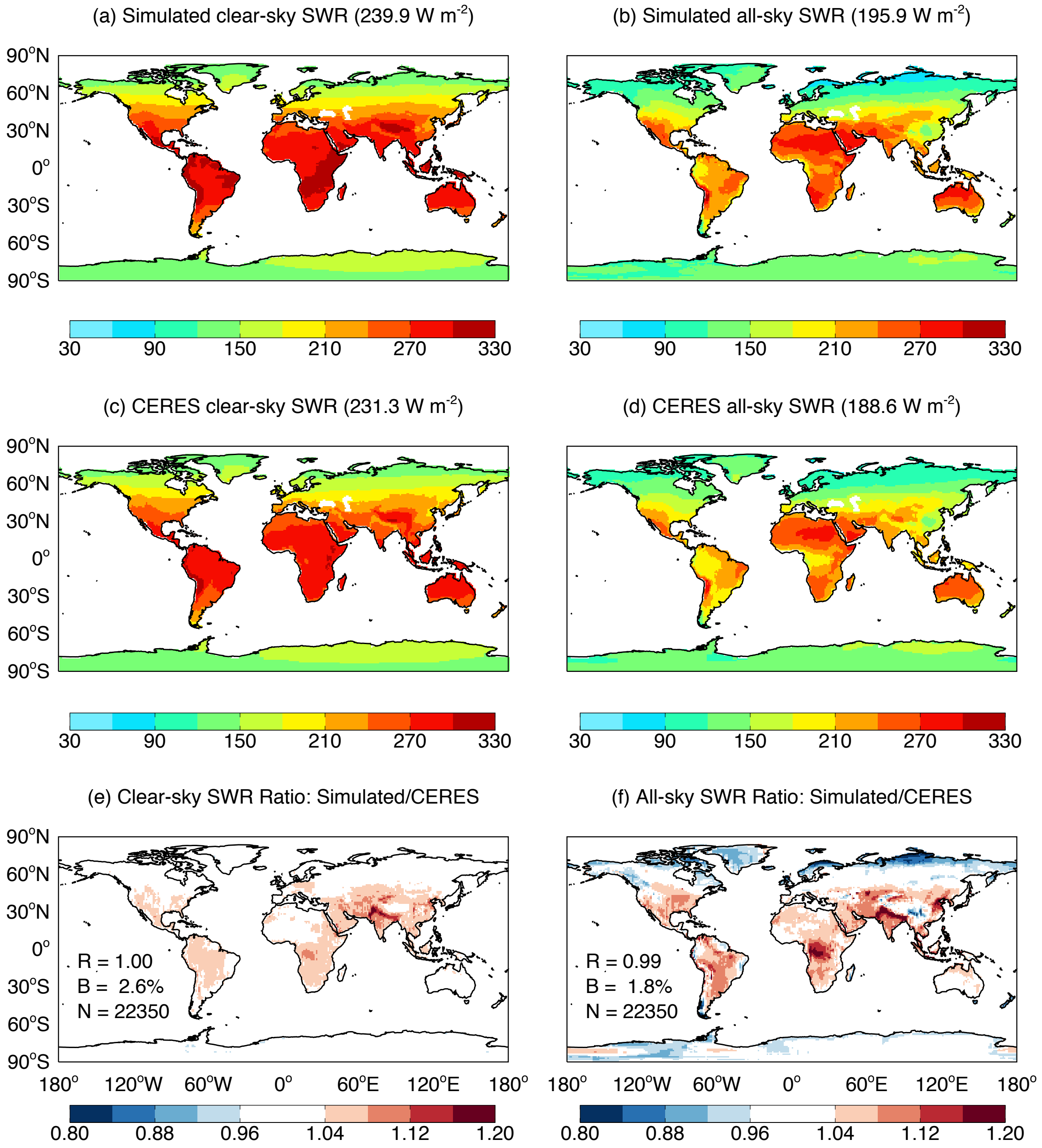

Supplementary Figure 14 | Evaluation of simulated shortwave radiation by CRM model. Results shown are annual mean surface downward shortwave radiation at (a, c) clear-sky and (b, d) all-sky conditions between (a, b) simulations, (c, d) observations, and (e, f) the simulation-to-observation ratio averaged for the period of 2001-2011. Simulations are performed with the Column Radiation Model (CRM), which is driven with hourly 1°×1° meteorological forcings from MERRA and cloud profiles from the SYN1deg product of CERES[2]. Observations are adopted from the CERES SYN1deg datasets. Global areal weighted shortwave radiation is shown on the title brackets. The correlation coefficient (R) and relative biases (B) are shown in the bottom figures with indication of total land grid number (N) used for statistics.

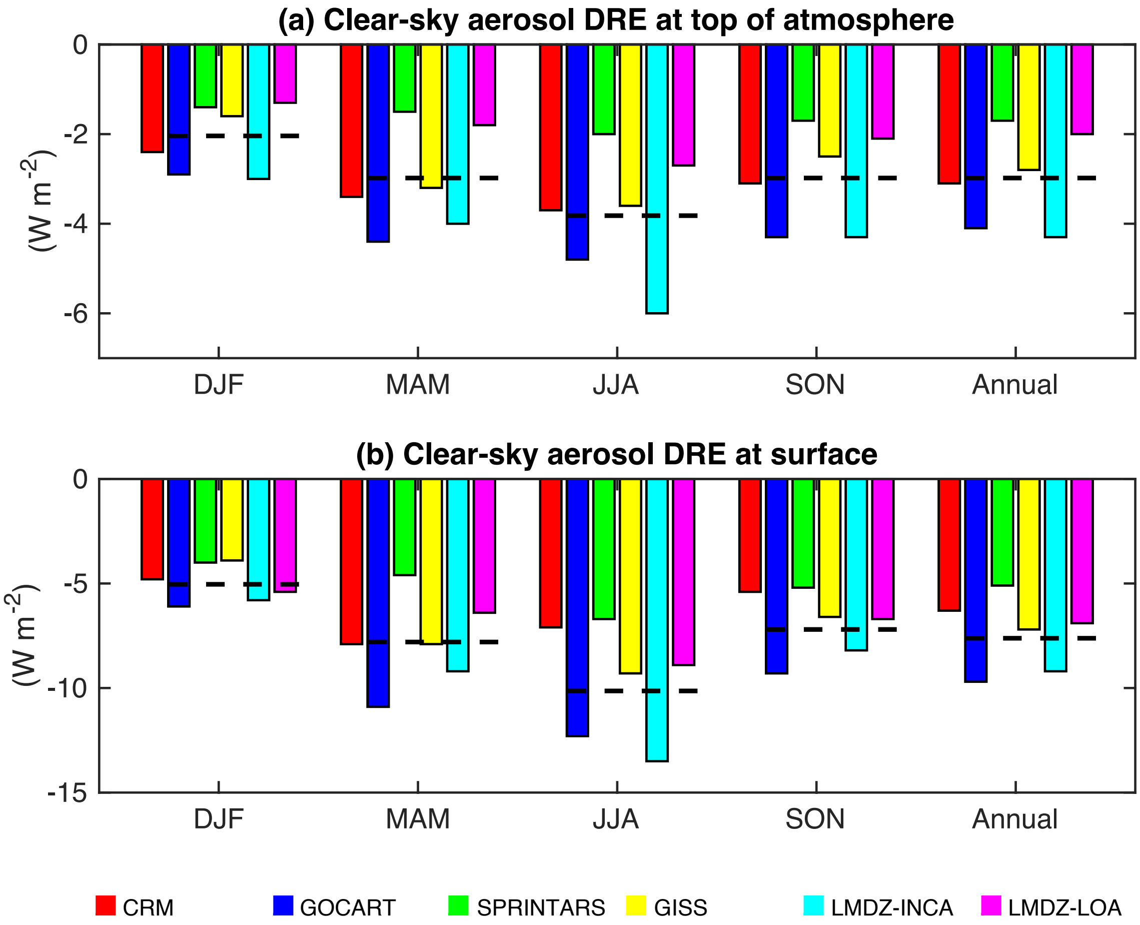

Supplementary Figure 15 | Comparison of simulated aerosol direct radiative effect (DRE) with other models. Results shown are aerosol DRE at (a) top of atmosphere and (b) surface between CRM and other models. Comparisons are performed for boreal winter (December-February), spring (March-May), summer (June-August), autumn (September-November), and annual average. Black dashed lines are the averages of estimates from other models. Different colors indicate different models, the details of which have been explained in Yu, et al. [6].

Supplementary References

1 Sitch, S., Cox, P. M., Collins, W. J. & Huntingford, C. Indirect radiative forcing of climate change through ozone effects on the land-carbon sink. *Nature* **448**, 791-794 (2007).

2 Rutan, D. A. et al. CERES Synoptic Product: Methodology and Validation of Surface Radiant Flux. *Journal of Atmospheric and Oceanic Technology* **32**, 1121-1143 (2015).

3 Weedon, G. P. et al. The WFDEI meteorological forcing data set: WATCH Forcing Data methodology applied to ERA-Interim reanalysis data. *Water Resources Research* **50**, 7505-7514 (2014).

4 Jung, M., Reichstein, M. & Bondeau, A. Towards global empirical upscaling of FLUXNET eddy covariance observations: validation of a model tree ensemble approach using a biosphere model. *Biogeosciences* **6**, 2001-2013 (2009).

5 Sofen, E. D. et al. Gridded global surface ozone metrics for atmospheric chemistry model evaluation. *Earth Syst Sci Data* **8**, 41-59 (2016).

6 Yu, H. et al. A review of measurement-based assessments of the aerosol direct radiative effect and forcing. *Atmos. Chem. Phys.* **6**, 613-666 (2006).

References for Supplementary Data 1 [1-62]

1 Ainsworth, E. A. Rice production in a changing climate: a meta-analysis of responses to elevated carbon dioxide and elevated ozone concentration. *Global Change Biol* **14**, 1642-1650 (2008).

2 Akhtar, N. et al. Effects of ozone on growth, yield and leaf gas exchange rates of two Bangladeshi cultivars of wheat (Triticum aestivum L.). *Environ Pollut* **158**, 1763-1767 (2010).

3 Barnes, J. D. & Pfirrmann, T. The Influence of CO2 and O-3, Singly and in Combination, on Gas-Exchange, Growth and Nutrient Status of Radish (Raphanus-Sativus L). *New Phytol* **121**, 403-412 (1992).

4 Biswas, D. K. et al. Genotypic differences in leaf biochemical, physiological and growth responses to ozone in 20 winter wheat cultivars released over the past 60 years. *Global Change Biol* **14**, 46-59 (2008).

5 Biswas, D. K. et al. Impacts of methods and sites of plant breeding on ozone sensitivity in winter wheat cultivars. *Agr Ecosyst Environ* **134**, 168-177 (2009).

6 Calatayud, V. et al. Responses of evergreen and deciduous Quercus species to enhanced ozone levels. *Environ Pollut* **159**, 55-63 (2007).

7 Cardoso-Vilhena, J., Balaguer, L., Eamus, D., Ollerenshaw, J. & Barnes, J. Mechanisms underlying the amelioration of O-3-induced damage by elevated atmospheric concentrations of CO2. *J Exp Bot* **55**, 771-781 (2004).

8 Contran, N. & Paoletti, E. Visible foliar injury and physiological responses to ozone in Italian provenances of Fraxinus excelsior and Fornus. *The Scientific World Journal* **7**, 90-97 (2007).

9 Edwards, G. S., Wullschleger, S. D. & Kelly, J. M. Growth and Physiology of Northern Red Oak - Preliminary Comparisons of Mature Tree and Seedling Responses to Ozone. *Environ Pollut* **83**, 215-221 (1994).

10 Farage, P. K. & Long, S. P. The effects of O-3 fumigation during leaf development on photosynthesis of wheat and pea: An in vivo analysis. *Photosynth Res* **59**, 1-7 (1999).

11 Fares, S., Oksanen, E., Lannenpaa, M., Julkunen-Tiitto, R. & Loreto, F. Volatile emissions and phenolic compound concentrations along a vertical profile of Populus nigra leaves exposed to realistic ozone concentrations. *Photosynth Res* **104**, 61-74 (2010).

12 Feng, Z., Zeng, H., Wang, X., Zheng, Q. & Feng, Z. Sensitivity of Metasequoia glyptostroboides to ozone stress. *Photosynthetica* **46**, 463-465 (2008).

13 Feng, Z. Z., Kobayashi, K. & Ainsworth, E. A. Impact of elevated ozone concentration on growth, physiology, and yield of wheat (Triticum aestivum L.): a meta-analysis. *Global Change Biol* **14**, 2696-2708 (2008).

14 Feng, Z. Z. et al. Effects of ozone exposure on sub-tropical evergreen Cinnamomum camphora seedlings grown in different nitrogen loads. *Trees-Struct Funct* **25**, 617-625 (2011).

15 Feng, Z. Z., Pang, J., Kobayashi, K., Zhu, J. G. & Ort, D. R. Differential responses in two varieties of winter wheat to elevated ozone concentration under

fully open-air field conditions. *Global Change Biol* **17**, 580-591 (2011).
16 Fernandezbayon, J. M., Barnes, J. D., Ollerenshaw, J. H. & Davison, A. W. Physiological-Effects of Ozone on Cultivars of Watermelon (Citrullus-Lanatus) and Muskmelon (Cucumis-Melo) Widely Grown in Spain. *Environ Pollut* **81**, 199-206 (1993).
17 Fu, Y. et al. Effects of Elevated Ozone Concentration on Maize Photosynthesis and Grain Quality. *Acta Agriculturae Boreali-Sinica* **23**, 120-124 (2008).
18 Grantz, D. A., Vu, H. B., Tew, T. L. & Veremis, J. C. Sensitivity of Gas Exchange Parameters to Ozone in Diverse C-4 Sugarcane Hybrids. *Crop Sci* **52**, 1270-1280 (2012).
19 He, X. Y. et al. Changes in effects of ozone exposure on growth, photosynthesis, and respiration of Ginkgo biloba in Shenyang urban area. *Photosynthetica* **45** (2007).
20 Kellomaki, S. & Wang, K. Y. Effects of elevated O-3 and CO2 concentrations on photosynthesis and stomatal conductance in Scots pine. *Plant Cell Environ* **20**, 995-1006 (1997).
21 Kitao, M., Komatsu, M., Yazaki, K., Kitaoka, S. & Tobita, H. Growth overcompensation against O3 exposure in two Japanese oak species, Quercus mongolica var. crispula and Quercus serrata, grown under elevated CO2. *Environ Pollut* **206**, 133-141 (2015).
22 Kleier, C., Farnsworth, B. & Winner, W. Biomass, reproductive output, and physiological responses of rapid-cycling Brassica (Brassica rapa) to ozone and modified root temperature. *New Phytol* **139**, 657-664 (1998).
23 Kouterick, K. et al. Foliar injury, leaf gas exchange and biomass responses of black cherry (Prunus serotina ehrh.) half-sibling families to ozone exposure. *Environ Pollut* **107**, 117-126 (2000).
24 Kronfuss, G., Polle, A., Tausz, M., Havranek, W. M. & Wieser, G. Effects of ozone and mild drought stress on gas exchange, antioxidants and chloroplast pigments in current-year needles of young Norway spruce [Picea abies (L.) Karst.]. *Trees-Struct Funct* **12**, 482-489 (1998).
25 Kull, O. et al. Photosynthetic responses of aspen clones to simultaneous exposures of ozone and CO2. *Canadian Journal of Forest Research* **26**, 639-648 (1996).
26 Matyssek, R., Gunthardtgoerg, M. S., Keller, T. & Scheidegger, C. Impairment of Gas-Exchange and Structure in Birch Leaves (Betula-Pendula) Caused by Low Ozone Concentrations. *Trees-Struct Funct* **5**, 5-13 (1991).
27 Miller, J. E. et al. Ultraviolet-B Radiation and Ozone Effects on Growth, Yield, and Photosynthesis of Soybean. *J Environ Qual* **23**, 83-91 (1994).
28 Mulchi, C. L. et al. Growth and Physiological-Characteristics of Soybean in Open-Top Chambers in Response to Ozone and Increased Atmospheric Co2. *Agr Ecosyst Environ* **38**, 107-118 (1992).
29 Noble, R., Jensen, K. F., Ruff, B. S. & Loats, K. Response of Acer-Saccharum Seedlings to Elevated Carbon-Dioxide and Ozone. *Ohio J Sci* **92**, 60-62 (1992).
30 Oguntimehin, I., Eissa, F. & Sakugawa, H. Simultaneous ozone fumigation and

fluoranthene sprayed as mists negatively affected cherry tomato (Lycopersicon esculentum Mill). *Ecotox Environ Safe* **73**, 1028-1033 (2010).

31 Pang, J., Kobayashi, K. & Zhu, J. G. Yield and photosynthetic characteristics of flag leaves in Chinese rice (Oryza sativa L.) varieties subjected to free-air release of ozone. *Agr Ecosyst Environ* **132**, 203-211 (2009).

32 Paoletti, E., Nali, C. & Lorenzini, G. Early responses to acute ozone exposure in two Fagus sylvatica clones differing in xeromorphic adaptations: Photosynthetic and stomatal processes, membrane and epicuticular characteristics. *Environ Monit Assess* **128**, 93-108 (2007).

33 Pellegrini, E., Francini, A., Lorenzini, G. & Nali, C. PSII photochemistry and carboxylation efficiency in Liriodendron tulipifera under ozone exposure. *Environ Exp Bot* **70**, 217-226 (2011).

34 Power, S. A. & Ashmore, M. R. Responses of fen and fen-meadow communities to ozone. *New Phytol* **156**, 399-408 (2002).

35 Reiling, K. & Davison, A. W. Effects of Ozone on Stomatal Conductance and Photosynthesis in Populations of Plantago-Major L. *New Phytol* **129**, 587-594 (1995).

36 Retzlaff, W. A., Williams, L. E. & Dejong, T. M. The Effect of Different Atmospheric Ozone Partial Pressures on Photosynthesis and Growth of 9 Fruit and Nut Tree Species. *Tree Physiol* **8**, 93-105 (1991).

37 Robinson, J. M. & Britz, S. J. Tolerance of a field grown soybean cultivar to elevated ozone level is concurrent with higher leaflet ascorbic acid level, higher ascorbate-dehydroascorbate redox status, and long term photosynthetic productivity. *Photosynth Res* **64**, 77-87 (2000).

38 Singh, E., Tiwari, S. & Agrawal, M. Effects of elevated ozone on photosynthesis and stomatal conductance of two soybean varieties: a case study to assess impacts of one component of predicted global climate change. *Plant Biology* **11**, 101-108 (2009).

39 Soldatini, G. F., Nali, C., Guidi, L. & Lorenzini, G. Photosynthesis of Hedera canariensis var. azorica variegated leaves as affected by ozone. *Photosynthetica* **35**, 247-253 (1998).

40 Taylor, M. D., Sinn, J. P., Davis, D. D. & Pell, E. J. The impact of ozone on a salt marsh cordgrass (Spartina alterniflora). *Environ Pollut* **120**, 701-705 (2002).

41 Tjoelker, M., Volin, J., Oleksyn, J. & Reich, P. Interaction of ozone pollution and light effects on photosynthesis in a forest canopy experiment. *Plant Cell Environ* **18**, 895-905 (1995).

42 Tjoelker, M. G. & Luxmoore, R. J. Soil-Nitrogen and Chronic Ozone Stress Influence Physiology, Growth and Nutrient Status of Pinus-Taeda-L and Liriodendron-Tulipifera-L Seedlings. *New Phytol* **119**, 69-81 (1991).

43 Tjoelker, M. G., Volin, J. C., Oleksyn, J. & Reich, P. B. Light Environment Alters Response to Ozone Stress in Seedlings of Acer-Saccharum Marsh and Hybrid Populus L .1. In-Situ Net Photosynthesis, Dark Respiration and Growth. *New Phytol* **124**, 627-636 (1993).

44 Torsethaugen, G., Pitcher, L. H., Zilinskas, B. A. & Pell, E. J. Overproduction

of ascorbate peroxidase in the tobacco chloroplast does not provide protection against ozone. *Plant Physiol* **114**, 529-537 (1997).

45 Volin, J. C., Reich, P. B. & Givnish, T. J. Elevated carbon dioxide ameliorates the effects of ozone on photosynthesis and growth: species respond similarly regardless of photosynthetic pathway or plant functional group. *New Phytol* **138**, 315-325 (1998).

46 von Tiedemann, A. & Firsching, K. H. Interactive effects of elevated ozone and carbon dioxide on growth and yield of leaf rust-infected versus non-infected wheat. *Environ Pollut* **108**, 357-363 (2000).

47 Wahid, A. Productivity losses in barley attributable to ambient atmospheric pollutants in Pakistan. *Atmos Environ* **40**, 5342-5354 (2006).

48 Wahid, A., Ahmad, S. S., Butt, Z. A. & Ahmad, M. Exploring the Hidden Threat of Gaseous Pollutants Using Rice (Oryza Sativa L.) Plants in Pakistan. *Pak J Bot* **43**, 365-382 (2011).

49 Watanabe, M., Umemoto-Yamaguchi, M., Koike, T. & Izuta, T. Growth and photosynthetic response of Fagus crenata seedlings to ozone and/or elevated carbon dioxide. *Landsc Ecol Eng* **6**, 181-190 (2010).

50 Wei, C. et al. Influence of light fleck and low light on foliar injury and physiological responses of two hybrid poplar clones to ozone. *Environ Pollut* **130**, 215-227 (2004).

51 Welfare, K., Flowers, T. J., Taylor, G. & Yeo, A. R. Additive and antagonistic effects of ozone and salinity on the growth, ion contents and gas exchange of five varieties of rice (Oryza sativa L). *Environ Pollut* **92**, 257-266 (1996).

52 Wiese, C. B. & Pell, E. J. Influence of ozone on transgenic tobacco plants expressing reduced quantities of Rubisco. *Plant Cell Environ* **20**, 1283-1291 (1997).

53 Wittig, V. E., Ainsworth, E. A. & Long, S. P. To what extent do current and projected increases in surface ozone affect photosynthesis and stomatal conductance of trees? A meta-analytic review of the last 3 decades of experiments. *Plant Cell Environ* **30**, 1150-1162 (2007).

54 Xu, S. et al. Differential sensitivity of four urban tree species to elevated O3. *Urban Forestry & Urban Greening* **14**, 1166-1173 (2015).

55 Yan, K. et al. Responses of photosynthesis, lipid peroxidation and antioxidant system in leaves of Quercus mongolica to elevated O3. *Environ Exp Bot* **69**, 198-204 (2010).

56 Yonekura, T., Dokiya, Y., Fukami, M. & Izuta, T. Effects of ozone and/or soil water stress on growth and photosynthesis of Fagaus crenata seedlings. *Water Air Soil Poll* **130**, 965-970 (2001).

57 Yuan, X. Y. et al. Assessing the effects of ambient ozone in China on snap bean genotypes by using ethylenediurea (EDU). *Environ Pollut* **205**, 199-208 (2015).

58 Zeuthen, J., Mikkelsen, T., Paludan-Muller, G. & Ro-Poulsen, H. Effects of increased UV-B radiation and elevated levels of tropospheric ozone on physiological processes in European beech (Fagus sylvatica). *Physiol Plantarum* **100** (1997).

59 Zhang, W. et al. Effects of ozone exposure on growth and photosynthesis of the seedlings of Liriodendron chinense (Hemsl.) Sarg, a native tree species of subtropical China. *Photosynthetica* **49**, 29-36 (2011).
60 Zhang, W. W., Feng, Z. Z., Wang, X. K. & Niu, J. F. Responses of native broadleaved woody species to elevated ozone in subtropical China. *Environ Pollut* **163**, 149-157 (2012).
61 Zheng, Y., Zhang, J., Wu, R., Zhao, Z. & Hu, C. Effects of ozone stress on photosynthesis and physiological characteristics of winter wheat in northern China. *Journal of Agro-Environment Science* **29**, 1429-1436 (2010).
62 Zouzoulas, D., Koutroubas, S. D., Vassiliou, G. & Vardavakis, E. Effects of ozone fumigation on cotton (Gossypium hirsutum L.) morphology, anatomy, physiology, yield and qualitative characteristics of fibers. *Environ Exp Bot* **67**, 293-303 (2009).
