# Supplementary material for: Fire air pollution reduces global terrestrial productivity
Source: Nat Commun. 2018 Dec 21;9:5413. doi: 10.1038/s41467-018-07921-4 (PMC6303378; doi:10.1038/s41467-018-07921-4)
Supplement: Supplementary file 3 — Description of Additional Supplementary Files [file 41467_2018_7921_MOESM3_ESM.pdf]

Description of Additional Supplementary Files

File Name: Supplementary Data 1
Description: Summary of measurement data for O_3 effects on plant photosynthesis from literatures
